# Higher odds of gestational diabetes among women with multiple pregnancies: a nationwide register-based cohort study in Finland

**DOI:** 10.1007/s00592-022-01984-y

**Published:** 2022-10-11

**Authors:** Matias Vaajala, Rasmus Liukkonen, Ville Ponkilainen, Maiju Kekki, Ville M. Mattila, Ilari Kuitunen

**Affiliations:** 1 Faculty of Medicine and Life Sciences, University of Tampere, Tampere, Finland; 2 Department of Surgery, Central Finland Central Hospital Nova, Jyväskylä, Finland; 3 Department of Obstetrics and Gynecology, Tampere University Hospital, Tampere, Finland; 4 Center for Child, Adolescent and Maternal Health Research, Faculty of Medicine and Health Technology, Tampere University, Tampere, Finland; 5 Department of Orthopaedics and Traumatology, Tampere University Hospital Tampere, Tampere, Finland; 6 Department of Pediatrics, Mikkeli Central Hospital, Mikkeli, Finland; 7 Institute of Clinical Medicine and Department of Pediatrics, University of Eastern Finland, Kuopio, Finland

**Keywords:** Multiple pregnancy, Gestational diabetes, GDM, Twin pregnancy

## Abstract

**Introduction:**

The association between multiple pregnancies and the risk of gestational diabetes mellitus (GDM) has been moderately studied. The aim of this study is to evaluate whether women with multiple pregnancies are at a higher risk of developing GDM using nationwide high-quality registers.

**Materials and methods:**

In this retrospective cohort study, data from the National Medical Birth Register (MBR) was used to evaluate the odds of GDM as a result of multiple pregnancies. We included all pregnancies with a tested GDM recorded in the MBR between 2004 and 2018. A total of 397,810 pregnancies were included in this study. Logistic regression model was used to assess the odds for GDM among multiple pregnancies, when compared to singleton pregnancies. Odds ratios (ORs) and adjusted odds ratios (aORs) with 95% confidence intervals (CIs) between the groups were compared. The model was adjusted with maternal BMI and in vitro fertilisation (IVF) treatments.

**Results:**

A total of 5825 multiple pregnancies and a tested GDM were observed. In the control group, there were 391,985 singleton pregnancies with a tested GDM. Of these, 1791 (30.7%) multiple pregnancies were associated with a diagnosis of GDM. GDM was more common among women with multiple pregnancies (30.7 vs. 25.9%, aOR 1.28; CI 1.21–1.36).

**Conclusion:**

The results of this study show that women with multiple pregnancies have a higher odds of developing GDM and should be monitored to prevent the development of GDM.

**Supplementary Information:**

This article belongs in the Topical Collection "Pregnancy and Diabetes" managed by Antonio Secchi and Marina Scavini. The online version contains supplementary material available at 10.1007/s00592-022-01984-y.

## Introduction

The association between multiple pregnancies and the risk of gestational diabetes mellitus (GDM) has been moderately studied. However, the results of previous research have been contradictory [1, 2]. A 2003 study with a small patient sample did not observe any increases in the rates of GDM among women with twin pregnancies [1]. A 2009 study that analysed a relatively large cohort found that twin pregnancies were associated with an approximately twofold increase in the risk for developing GDM [2]. A Finnish study found that the rates of GDM among women with twin pregnancies increased from 1987 to 2004 [3]. Despite these studies, large nationwide studies on the effects of multiple pregnancies on the development of GDM in women are lacking.

The increased production of human placental lactogen in twin pregnancies causes insulin intolerance [4]. In association with other factors, such as weight gain, maternal age and body mass index (BMI), this could lead to gestational diabetes [4]. Based on our hypothesis and previous study results, multiple pregnancies might be a risk factor for the development of GDM. Hence, the aim of this study is to evaluate whether women with multiple pregnancies are at a higher risk of developing GDM using nationwide high-quality registers.

## Materials and methods

In this nationwide retrospective register-based cohort study, data from the National Medical Birth Register (MBR) were used to evaluate the odds of GDM as a result of multiple pregnancies. The MBR is maintained by the Finnish Institute for Health and Welfare. The study period was from 1 January 2004 to 31 December 31 2018.

**Fig. 1:**
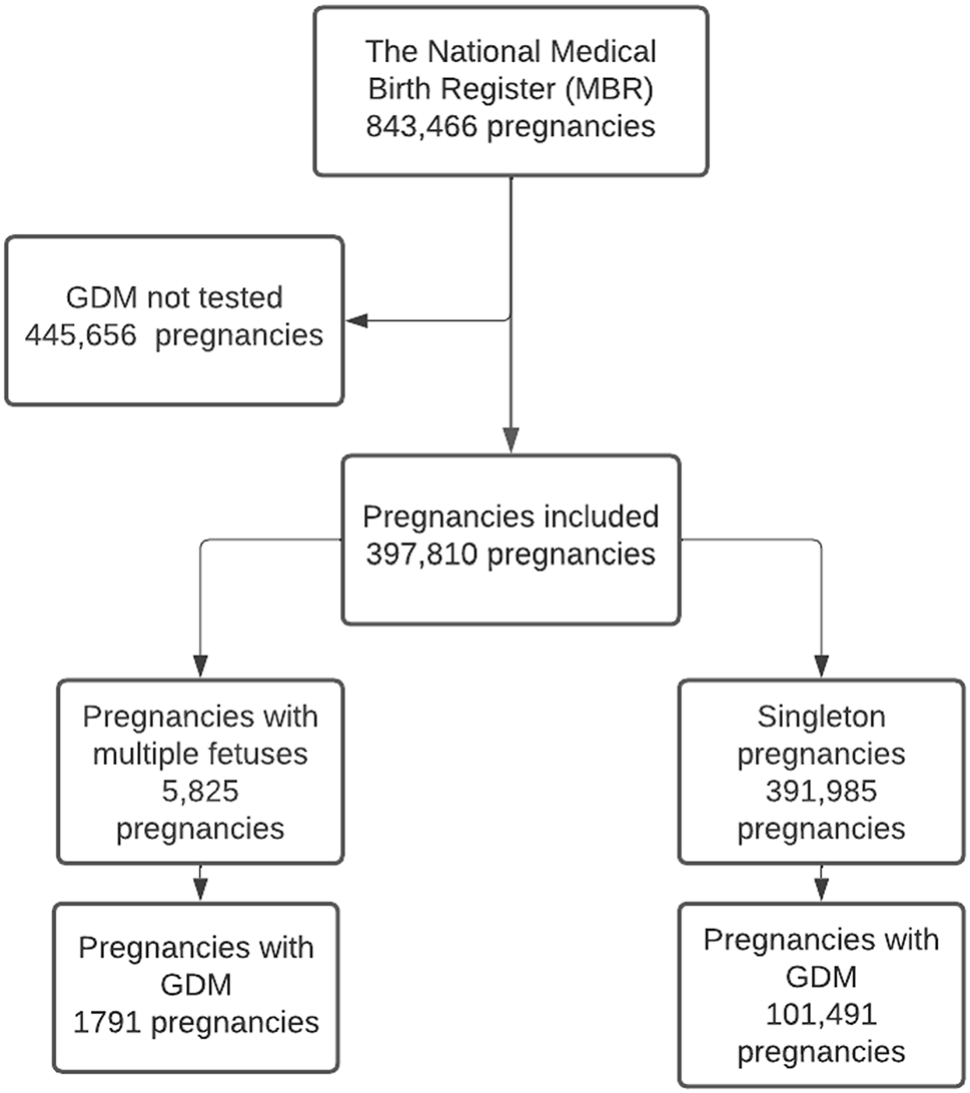
Flow chart depicting the process used to divide the study population into groups. Multiple pregnancies were compared to singleton pregnancies

The MBR contains data on pregnancies, delivery statistics and the perinatal outcomes of all births with a birthweight of ≥ 500 g or a gestational age of ≥ 22^+0^ weeks. The MBR has high coverage and quality (the current coverage is nearly 100%). In Finland, GDM is diagnosed in the second trimester with a 2 h 75 g oral glucose tolerance test. We included all pregnancies with a tested GDM recorded in the MBR between 2004 and 2018. A total of 397,810 pregnancies were included in this study. Among multiple pregnancies (*N* = 5825), a total of 48 pregnancies featured 3 foetuses, and two featured 4 foetuses. The process used to form the study groups is shown as a flow chart in Fig. 1.

Continuous variables were interpreted as means with standard deviations (SDs) or as a median with an interquartile range (IQR) based on the distribution of the data. The categorical variables are presented as absolute numbers and percentages. Student’s t-test, Mann–Whitney U-tests and Chi-squared tests were used to compare the groups. A logistic regression model was used to assess the primary outcome. The exposure variable was multiple pregnancy (yes/no), and the primary outcome was whether GDM was diagnosed. Odds ratios (ORs) and adjusted odds ratios (aORs) with 95% confidence intervals (CIs) between the groups were compared. The model was adjusted with the categorised BMIs of the mothers and in vitro fertilisation (IVF) treatments, as maternal obesity is known to be a risk factor for GDM, and IVF has been found to be a possible risk factor for GDM [5]. The BMI of the mother was categorised using the World Health Organization’s (WHO) classifications of underweight (BMI < 18.5), normal weight (BMI 18.5–25.0), pre-obesity (BMI 25.0–30.0), obesity class I (BMI 30–35), obesity class II (BMI 35–40) and obesity class III (BMI over 40). Due to change of screening methods of GDM in 2008, we performed a sensitivity analysis for the main analysis using time periods before and after the year 2008 (1.1.1998–31.12.2008 and 1.1.2008–31.12.2018) separately (Supplementary table 1).

## Results

A total of 5825 multiple pregnancies and a tested GDM were observed. In the control group, there were 391,985 singleton pregnancies with a tested GDM. Of these, 1791 (30.7%) multiple pregnancies were associated with a diagnosis of GDM. In the control group, there were 101,491 (25.9%) singleton pregnancies associated with a diagnosis of GDM (*p* < 0.001). Women having multiple pregnancies were older than women having singleton pregnancies (31.6 years vs. 30.5 years, *p* < 0.001). GDM was more common among women with multiple pregnancies (30.7 vs. 25.9%, aOR 1.28; CI 1.21–1.36) (Table 1). The odds for GDM among multiple pregnancies were also higher in sensitivity analyses (supplementary table 1).

**Table 1:** Background information on study groups, odds ratios (OR) and adjusted odds ratios (aOR) with 95% confidence intervals (CI).

Total number	Multiple pregnancies		Singleton pregnancies	
	5825		391,985	
	*n*	%	*n*	%
Age at the time of pregnancy (mean, sd)	31.6 (5.2) years		30.5 (5.3) years	
Diagnosed gestational diabetes	1791	30.7	101 491	25.9
*BMI*				
Underweight < 18.5	88	1.5	6768	0.7
Normal weight	2641	45.3	172,856	44.0
Pre-obesity	1779	30.5	125,874	32.0
Obesity class I	805	13.8	53,754	13.7
Obesity class II	318	5.5	19,156	5.0
Obesity class III	112	1.9	8497	2.2
Unknown	82	1.4	5080	1.3
Pregnancy stated with IVF	27	0.5	5798	1.5
*Logistic regression*				
	OR (CI)		aOR (CI)	
	1.27 (1.20–1.35)		1.28 (1.21–1.36)	

Multiple pregnancies were compared with singleton pregnancies. The model was adjusted with the BMI class of the mother at the beginning of pregnancy and IVF (in vitro fertilisation)

## Discussion

The main finding of this study is that the odds of GDM were higher among women with multiple pregnancies. A study on the effects of GDM found that the odds of GDM were over two times higher, but our nationwide results suggest that the odds of GDM are not that high [2]. However, unlike in some of the older studies, which found no difference between multiple and singleton pregnancies, we still found that multiple pregnancies markedly increased the odds of GDM. After adjusting the model using the maternal BMI classes, the odds of GDM increased, meaning that a higher BMI is not the only factor that can explain the higher rate of GDM in women with multiple pregnancies. Clinicians should acknowledge the results of this study to prevent the development of GDM in women with multiple pregnancies, as GDM has many negative effects on the health of a foetus.

The strengths of our study are the large nationwide register data used and the long study period, which allowed us to analyse the effects of multiple pregnancies using larger datasets than in previous studies. The register data used in our study are routinely collected in structured forms using national instructions, which ensures good coverage (over 99%) and reduces possible reporting and selection biases. A possible limitation of this study is that the screening methods for GDM changed after 2008 to comprehensive screening, meaning that the GDM testing rates increased towards the end of the study period. However, after performing sensitivity analyses (supplementary table 1), we believe that this should not have important effect on the results of this study.

In conclusion, the results of this study show that women with multiple pregnancies have a higher odds of developing GDM and should be monitored to prevent the development of GDM.

## Supplementary Information

Below is the link to the electronic supplementary material.

Supplementary file1 (PDF 34 kb)
