# Supplementary material for: Higher odds of gestational diabetes among women with multiple pregnancies: a nationwide register-based cohort study in Finland
Source: Acta Diabetol. 2022 Oct 11;60(1):127–30. doi: 10.1007/s00592-022-01984-y (PMC9813100; doi:10.1007/s00592-022-01984-y)
Supplement: Supplementary file 1 — Supplementary file1 (PDF 34 kb) [file 592_2022_1984_MOESM1_ESM.pdf]

Supplementary table 1: Sensitivity analysis with different time periods, as the screening methods changed to comprehensive screening in 2008. Time periods of 2004-2007 and 2008-2018 were analysed separately. Odds ratios (OR) and adjusted odds ratios (aOR) with 95% confidence intervals (CI). Multiple pregnancies were compared with singleton pregnancies. The model was adjusted with the BMI class of the mother at the beginning of pregnancy and IVF (in vitro fertilization).

Logistic regression		
	OR (CI)	aOR (CI)
Years 2004-2007	1.24 (1.09 – 1.41)	1.25 (1.09 – 1.43)
Years 2008-2018	1.28 (1.21 – 1.37)	1.28 (1.21 – 1.36)
